# Comparative Transcriptomic Analysis on the Effect of Sesamol on the Two-Stages Fermentation of *Aurantiochytrium* sp. for Enhancing DHA Accumulation

**DOI:** 10.3390/md22080371

**Published:** 2024-08-16

**Authors:** Xuewei Yang, Liyang Wei, Shitong Liang, Zongkang Wang, Shuangfei Li

**Affiliations:** 1 Guangdong Technology Research Center for Marine Algal Bioengineering, Guangdong Key Laboratory of Plant Epigenetics, College of Life Sciences and Oceanography, Shenzhen University, Shenzhen 518060, China; 2200251002@szu.edu.cn (L.W.); 2060251019@szu.edu.cn (S.L.); 2 Ecological Fertilizer Research Institute, Shenzhen Batian Ecological Engineering Co., Ltd., Shenzhen 518057, China; wangzongkang712@163.com

**Keywords:** *Aurantiochytrium*, DHA, thraustochytrid, two-stage fermentation, transcriptomics

## Abstract

*Aurantiochytrium* is a well-known long-chain polyunsaturated fatty acids (PUFAs) producer, especially docosahexaenoic acid (DHA). In order to reduce the cost or improve the productivity of DHA, many researchers are focusing on exploring the high-yield strain, reducing production costs, changing culture conditions, and other measures. In this study, DHA production was improved by a two-stage fermentation. In the first stage, efficient and cheap soybean powder was used instead of conventional peptone, and the optimization of fermentation conditions (optimal fermentation conditions: temperature 28.7 °C, salinity 10.7‰, nitrogen source concentration 1.01 g/L, and two-nitrogen ratio of yeast extract to soybean powder 2:1) based on response surface methodology resulted in a 1.68-fold increase in biomass concentration. In the second stage, the addition of 2.5 mM sesamol increased the production of fatty acid and DHA by 93.49% and 98.22%, respectively, as compared to the optimal culture condition with unadded sesamol. Transcriptome analyses revealed that the addition of sesamol resulted in the upregulation of some genes related to fatty acid synthesis and antioxidant enzymes in *Aurantiochytrium*. This research provides a low-cost and effective culture method for the commercial production of DHA by *Aurantiochytrium* sp.

## 1. Introduction

Docosahexaenoic acid (DHA, C22:6) belongs to omega-3 long-chain polyunsaturated fatty acids (PUFAs), and it is an essential structural element of the brain, retina, and neuron cell membrane [1]. DHA has demonstrably beneficial effects on depression [2], lowering cardiovascular risk [3], suppressing inflammation [4] and atherosclerosis [5], improving nervous system and retina development [6], and decreasing cancer risk [7]. The human body is unable to produce DHA; hence, it must come from food. Since marine fish and fish oil is cheap and contains a lot of DHA, it is generally thought to be the greatest source of DHA in the human diet [8]. However, marine pollution and overfishing limit the increasing demand for DHA [9]. Creating substitute sources to fulfill the demand for DHA is a significant task. Marine oleaginous microorganisms, as the original source of DHA production in marine fish, have garnered significant attention globally in the search for the sustainable production of DHA because of their potential applications in the biopharmaceutical and nutraceutical sectors [10,11]. The single-celled eukaryotic thraustochytrid, which includes the *Thraustochytrium*, *Aurantiochytrium*, and *Schizochytrium*, is currently the main focus of research. These organisms are typically found in marine environments and are capable of synthesizing large levels of lipids and carotenoids. Among them, it has been previously documented that *Aurantiochytrium* sp. can accumulate significant lipid content, particularly DHA [12,13].

Thraustochytrids are a prospective new market for DHA synthesis, and increasing DHA production is now a focus of intense study. The optimization of fermentation conditions (such as varying the source of nitrogen, C/N ratio, temperature, salinity etc.) as a crucial tactic to boost biomass and fatty acids production has been an important focus for thraustochytrid biotechnological research [14]. Fermentation costs are largely influenced by the carbon and nitrogen substrates used, and since nitrogen sources are often more expensive than carbon sources, less expensive options are being considered. Both organic (such as soybean meal hydrolysate, yeast extract, peptone, and monosodium glutamate) and inorganic (such as nitrate and ammonium) nitrogen can be used by thraustochytrids [15]. To reduce production costs, traditional nitrogen sources may be replaced with agricultural products and the food industry by-products [16]. The two-stage strategy, which divides the biomass growth phase from the lipid accumulation phase, is an efficient cultivation system [17]. The purpose of the first stage is to produce as much biomass as possible when nutrition is adequate, while the second stage—which is typically nitrogen-starved but has an excess of the carbon source—is meant to accumulate lipids [18].

An increasing number of research studies have shown that incorporating antioxidants such as vitamin C, plant hormone, and melatonin into the culture medium could enhance the ability of oleaginous microorganisms to produce lipids [19]. In a prior investigation, the utilization of *Dioscorea zingiberensis*’s phenolic-rich starch saccharification liquid markedly enhanced *Schizochytrium* sp.’s DHA yield and antioxidant capability [20]. One of the primary naturally occurring phenols in sesame, sesamol, has a potent antioxidant capacity and enhancement of radical scavenging [21], and it is frequently employed as an inexpensive, non-toxic antioxidant to stop lipid peroxidation in food and medicine. Sesamol supplementation has shown increased DHA production in *Schizochytrium*. The addition of 1 mM sesamol exogenously to the fermentation medium increased *Schizochytrium* sp. H016’s yield of DHA and lipids by 53.52% and 78.30%, respectively [22].

This study optimizes the type of nitrogen source in the culture medium in terms of cost and utilization efficiency. According to the four factors of temperature, salinity, nitrogen source concentration and the ratio of two nitrogen sources (yeast extract and soybean powder), the best level was selected by a one-factor test. And then the culture conditions of four factors and three levels were optimized to enhance the biomass yield of *Aurantiochytrium* sp. DECR-KO (2,4-dienyl-CoA reductase-knockout) [23] on response surface methodology (RSM). The effects of various sesamol concentrations on the biomass, lipid accumulation, and the synthesis of fatty acids of *Aurantiochytrium* sp. DECR-KO were examined in this study. This study elucidated the possible mechanisms of lipid metabolism regulation through antioxidant supplementation through transcriptome analysis.

## 2. Results

### 2.1. Screening of Different Nitrogen Source Components

The N content of the six nitrogen source components was determined by an elemental analyzer, and the results are shown in Table S1. All six nitrogen sources had more than 10% of N content, among which the peptone contained the highest N, up to 13.35%. In order to reflect the types of nitrogen sources that can be efficiently utilized by *Aurantiochytrium* sp. DECR-KO, we added the six nitrogen sources at an addition rate of 2.5 g/L into the M4 medium without yeast extract and peptone to examine the effects of various nitrogen source components on the biomass concentration of *Aurantiochytrium* sp. DECR-KO.

**Figure 1:**
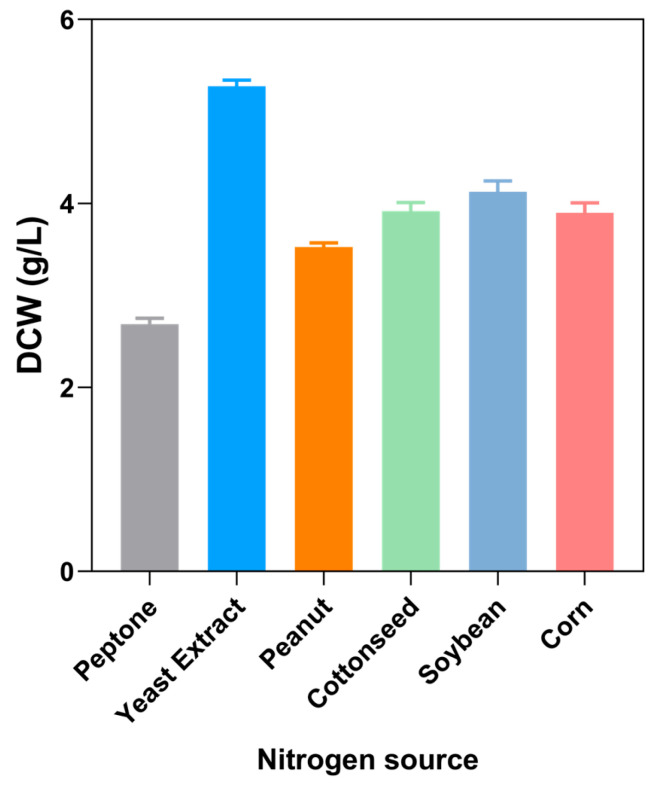
Effects of various nitrogen sources on the biomass concentration of *Aurantiochytrium* sp. DECR-KO. Cell dry weight (DCW) was obtained by culture for 65 h with 2.5 g/L different nitrogen source.

The results of the initial screening of nitrogen source types are shown in Figure 1. Among the biomass concentration results from the 65 h incubation under the same nitrogen source addition concentration, the best biomass concentration was obtained from the culture by yeast extract, which amounted to 5.28 ± 0.07 g/L, and the second one was obtained from the soybean powder, with the obtained biomass concentration of 4.13 ± 0.12 g/L. The lowest biomass concentration obtained in culture was peptones, only 2.69 ± 0.06 g/L, which was 0.51 times that of yeast extract and 0.65 times that of soybean powder. Combining the effects of the six nitrogen sources on the biomass concentration of *Aurantiochytrium* sp. DECR-KO and its market price, the low-cost and high-efficiency soybean powder was selected to replace the original high-cost and low-utility peptone. The two nitrogen sources, yeast extract and soybean powder, will be used in subsequent studies for the compounding and optimization of culture conditions.

### 2.2. Response Surface Methodology (RSM) to Optimize the Biomass Yield

**Figure 2:**
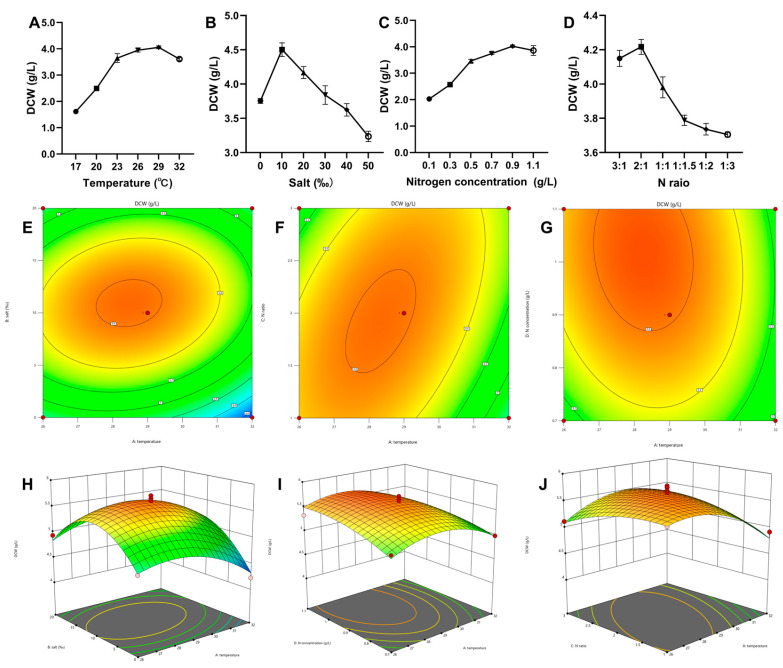
(**A**) The effects of six different temperature levels; (**B**) the effects of six different salinity levels; (**C**) the effects of six different nitrogen sources; (**D**) the effect of the ratio of two nitrogen sources (yeast extract: soybean powder). Contour plots showing the effect of (**E**) temperature and salt (**F**), temperature and nitrogen ratio (**G**), temperature and nitrogen source concentration to dry cell weight (DCW). Response surface plots show the effect of (**H**) temperature and salt, (**I**) temperature and N ratio, (**J**) temperature and nitrogen source concentration to DCW.

Using a single-factor experiment and an RSM central composite design based on the fermentation factors of *Aurantiochytrium* sp. DECR-KO, the fermentation factors were optimized in this study to increase the biomass concentration on the first stage. The results show that the temperature of 26 °C, salinity 10‰, nitrogen source concentration 0.90 g/L and N ratio 2:1 (yeast extract:soybean powder) were the optimal single factor levels for biomass concentration (Figure 2A–D). The Box–Behnken experiment designed a total of 29 sites, including 5 central sites and 24 factorial points (Table 1). The central experimental point is the central point of the partition, which can be used as the calibration of the points. With cell dry weight (DCW) as the response value (Y), and temperature (A), salinity (B), the ratio of two nitrogen sources (C), and the nitrogen source concentration (D) as independent variables, the response surface data as follows:

DCW (g/L) = 5.61313 − 0.12102 × A + 0.134542 × B + 0.0265687 × C + 0.134915 × D + 0.121345 × AB + 0.21728 × AC − 0.0554881 × AD − 0.309067 × A^2^ − 0.604891 × B^2^ − 0.14348 × C^2^ − 0.128604 × D^2^

The model was built using the software Design Expert 12 to obtain a multiple quadratic regression response surface model for biomass yield, and the model obtained from fitting the experimental results was subjected to ANOVA (Table 2) to verify the usability of the regression model. The ANOVA yielded a model with a *p*-value of <0.0001, which is extremely significant; where the lack of fit had a *p*-value of 0.1640, which is greater than 0.05, and the lack of fit was not significant. These two items indicate that the model obtained by fitting this response surface analysis is accurate in its predictions. The contour plots and response surface curves of the interaction terms were obtained by simulation with Design Expert 12 software (Figure 2E–J). According to the *F*-value of each factor, the order of influence on biomass concentration of *Aurantiochytrium* sp. DECR-KO was D (nitrogen concentration) > B (salinity) > A (temperature) > C (ratio of two nitrogen sources). In this experiment, the differences in the effects on biomass concentration were extremely significant for the secondary term B^2^, highly significant for the secondary term A^2^, and reached significance for the primary terms A, B, D, and the interaction term AC.

Meanwhile, the reliability analysis of the BBD model was also obtained through the fitting and analysis of the software. As shown in Table 2, the coefficient of variation in the model fitted in this experiment is 3.59, which is within the normal range. The regression coefficient R^2^ is 85.94% > 85%, which indicates that the equation created by the fitted model fits well. Adjusted R^2^ is the correction coefficient, and its value is 0.7684. Predicted R^2^ is the prediction coefficient, and its value is 0.5766. The difference between the correction coefficient and prediction coefficient is less than 0.2, which is within a reasonable range. The signal-to-noise ratio of this model is 10.919 according to “Adeq Precision”, which is greater than 4, indicating that the signal is sufficient and the model is reliable.

The culture conditions for the highest biomass yield were fitted by Design-Expert 12 software as follows: temperature 28.7 °C, salinity 10.7‰, nitrogen source concentration 1.01 g/L, and two-nitrogen ratio of yeast extract to soybean powder 2:1 (i.e., 11.62 g/L artificial sea salt, 3.16 g/L yeast extract, and 1.58 g/L water-soluble soybean powder). The predicted biomass yield that could be obtained under this optimal condition was 5.66 g/L of DCW. Three parallel experiments were conducted to validate under the predicted optimal culture conditions. The DCW results of the experiments were 5.56 g/L, 5.34 g/L, and 5.05 g/L, with an average value of 5.32 g/L, which was approximately 6% different from the predicted value.

**Figure 3:**
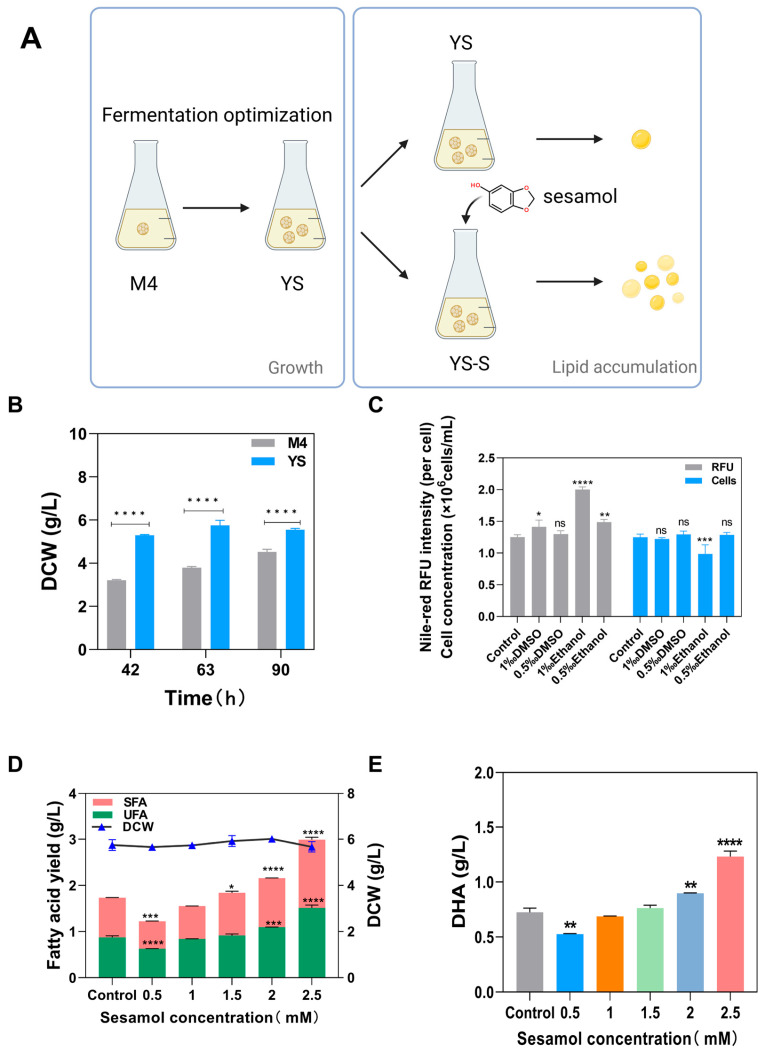
(**A**) Schematic diagram of enhancing lipid production in *Aurantiochytrium* sp. DECR-KO. (**B**) The difference of biomass concentration in YS and M4 culture after exponential phase. (**C**) Effects of DMSO and ethanol on cell concentration and neutral lipid content of *Aurantiochytrium* sp. DECR-KO at different volume concentrations. Solvent addition amount is expressed as volume concentration (*v*/*v*), and YS culture without adding any solvent was used as the control group. Cell concentration was shown as the number of cells per milliliter of culture medium, and neutral lipid content was represented by the relative fluorescence intensity of Nile red in each cell. (**D**,**E**): Effects of varying sesamol concentrations on DECR-KO strains’ fatty acid yield and DHA synthesis. M4: experimental group before fermentation optimization; YS: experimental group cultured with yeast extract and soybean powder after fermentation optimization; YS-S: experimental group YS treated with 2.5 mM sesamol. DHA: docosahexaenoic acid; UFAs: unsaturated fatty acids; SFAs: saturated fatty acids. ns: not significant, * *p* < 0.05, ** *p* < 0.01, *** *p* < 0.001, and **** *p* < 0.0001.

The experimental group which was cultured using response surface methodology was named YS (yeast extract–soybean powder, Figure 3A). As shown in Figure 3B, the biomass yields of the different culture methods of YS and M4 were investigated at three time points including the exponential growth period (42 h), stationary period (63 h), and decline period (90 h) of the culture [23]. It can be observed that the difference in biomass concentration of YS and M4 cultures at the mid-late stage of the culture decreased gradually with the gradually decreasing with increasing incubation time: at 42 h, the biomass concentration of the YS culture was 1.68 times more than that of the M4 culture; at 63 h, the biomass concentration of the YS culture was 1.51 times more than that of the M4 culture; and at 90 h, the biomass concentration of the YS culture was 1.23 times higher than that of the M4 culture. At the decline period of the *Aurantiochytrium* sp. DECR-KO, the biomass concentration of the M4 cultures was still rising, while the YS cultures declined, but the biomass yields obtained from the YS cultures were greater than those obtained from the M4 cultures throughout the mid-late phase of the culture.

### 2.3. Effect of Sesamol on the Fatty Acid Production Capacity of Aurantiochytrium sp. DECR-KO

As sesamol is insoluble in water, pre-experiments were carried out on both DMSO and ethanol in order to rule out the impact of the solvents on the biomass concentration and neutral lipid content of *Aurantiochytrium* sp. DECR-KO. There was no significant difference between the 0.5‰ (*v*/*v*) addition of DMSO and the control (Figure 3C), indicating that 0.5‰ of DMSO had no effect on the cell concentration of *Aurantiochytrium* sp. DECR-KO as well as the neutral lipid content. On the other hand, the neutral lipid content or cell concentration was significantly impacted by the volume ratio of 1‰ of DMSO and the ethanol of 0.5‰ and 1‰. Therefore, the subsequent sesamol will be solubilized using DMSO, and the volume ratio of the added solution will be controlled to be less than or equal to 0.5‰.

The addition of 0.5 mM to 2.5 mM sesamol had no visible effect on DCW compared to the control group (Figure 3D). The fatty acid yield of *Aurantiochytrium* sp. DECR-KO increased as the concentration of sesamol increased under 0.5–2.5 mM sesamol treatment (Figure 3D). However, in contrast to the control group, the fatty acid production decreased slightly when treated with low concentrations (0.5 to 1 mM) of sesamol, indicating that low concentrations of sesamol inhibited the production of fatty acids. When the concentration of sesamol treatment was greater than 1 mM, the fatty acid production was more than the control group. With the increase in the concentration of sesamol, the total fatty acid production (including saturated fatty acids and unsaturated fatty acids) also increased gradually. The DHA yield showed the same trend as the total fatty acid production (Figure 3E). Based on the results of the 0.5–2.5 mM sesamol treatments explored in this experiment, 2.5 mM was the optimal sesamol treatment concentration that most improved fatty acid yield. Compared to the control group, the 2.5 mM sesamol-treated group showed a 78.79% increase in total fatty acid yield and 69.83% increase in DHA yield.

### 2.4. Biomass Concentration and Fatty Production Analysis of Fermentation-Optimized

**Figure 4:**
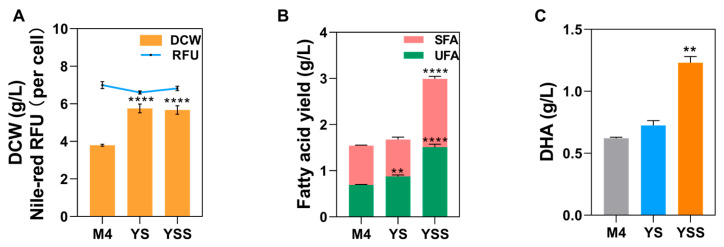
Difference of (**A**) biomass concentration, (**B**) fatty acids and (**C**) DHA yield in fermentation optimization experiments. ** *p* < 0.01 and **** *p* < 0.0001.

In order to better compare the optimization effect produced by fermentation optimization, the experimental group before fermentation optimization was named as M4, the experimental group using response surface methodology to optimize the biomass culture was named as YS (yeast extract and soybean powder), and the group treated with 2.5 mM sesamol was named as YSS (YS with sesamol treatment) (Figure 3A). Figure 4 displays the results of a comparison of the biomass concentration, fatty acid yield, and DHA yield of the three culture conditions. Compared with the M4 group, the biomass concentrations of the YS and YSS groups were increased by 50.06% and 49.85%, respectively. The addition of 2.5 mM sesamol treatment did not significantly affect the biomass concentration in the YS group during the stationary period (Figure 4A). Fatty acid production is shown in Figure 4B, and it can be seen that although the YS group increased the biomass concentration, its total fatty acid production was only increased by 8.23% compared to that of the M4 group. The addition of 2.5 mM sesamol increased the total fatty acid yield by 93.49% and 78.79% compared to the M4 and YS groups, respectively. A comparison of DHA yields among the three groups is shown in Figure 4C, which showed an increase of 16.71% and 98.22% in the YS and YSS groups, respectively, compared to the M4 group. The addition of 2.5 mM sesamol treatment to the experimental group increased DHA production by 69.83% compared to no sesamol addition.

### 2.5. Transcriptome Profiling of Aurantiochytrium sp. DECR-KO with Sesamol Treatment

The mechanism of *Aurantiochytrium* sp. DECR-KO’s response to sesamol treatment was ascertained by utilizing the Illumina RNA-seq method. There were 2677 differentially expressed genes (DEGs) in total identified (*p*-adjust < 0.05 and |log2FC| ≥ 1). A total of 1411 genes were upregulated and 1266 genes were downregulated in the YSS compared to the YS group. KEGG was used to examine the biological roles and interactions of the discovered DEGs. According to KEGG, notable enrichment pathways involved in lipid metabolism include fatty acid degradation and elongation, fatty acid metabolism, peroxisome and the biosynthesis of unsaturated fatty acids (Figure S1).

In oleaginous microorganisms, acetyl-CoA is an important central metabolite of carbon metabolism and one of the key precursors for fatty acid synthesis [24]. The process of glycolysis is one of the sources of acetyl coenzyme A. In glycolysis, 6-phosphofructokinase (PFK) is the rate-limiting enzyme, and PFK was upregulated by 1.07-fold in the YSS group (Table 3). Additionally, other key enzyme genes, including triosephosphate isomerase (TPI) and glyceraldehyde 3-phosphate dehydrogenase (GAPDH), were upregulated by 1.00-fold and 1.21-fold, respectively. In pyruvate metabolism, malate dehydrogenase was upregulated by 1.00-fold, which catalyzes the conversion of malate to pyruvate. Increasing the expression of the above genes results in more pyruvate production, and pyruvate produces acetyl-CoA through pyruvate dehydrogenase.

Acetyl-CoA carboxylase (ACC) catalyzes the conversion of acetyl-CoA to malonyl-CoA, a direct substrate for the synthesis of fatty acids, limiting the rate of fatty acid synthesis [25]. Acetyl-CoA carboxylase was upregulated by 0.81-fold in the YSS group. Malonyl-CoA:ACP transacylase (MCAT) was upregulated by 1.07-fold in the YSS group, converting malonyl-CoA to malonyl-ACP to initiate the elongation cycle. Concentrations of both SFA and UFA increased under sesamol treatment. In the fatty acid synthase (FAS) pathway, FAS was upregulated by 0.92-fold in the YSS group, which led to the accumulation of the SFA. In the polyketide synthase (PKS) pathway, 3-ketoacyl-CoA synthase (KS) and ketoreductase (KR) were upregulated by 0.68-fold and 1.33-fold. However, other co-catalyzing enzymes associated with the PKS pathway, dehydrase/isomerase (DH/I) and enoyl reductase (ER), were not identified in this study, which were similarly not identified in previous studies [26]. The above results indicated that fatty acid synthesis was enhanced under 2.5 mM sesamol treatment, which was in agreement with the experimental results. Except for fatty acid synthesis, fatty acid degradation is an important factor affecting lipid content. The fatty acid β-oxidation pathway is an important pathway for fatty acid degradation [24]. In the fatty acid β-oxidation pathway, acyl-CoA dehydrogenase (ACD), enoyl-CoA hydratase (ECH), 3-hydroxyacyl-CoA dehydrogenase (HADH), and 3-ketoacyl-CoA thiolase (KAT) were significantly more expressed in the YSS group than in the YS group (Table 3).

Superoxide dismutase (SOD) is one of the major intracellular enzymes that protects cells from oxidative damage [27]. SOD was upregulated 1.27-fold in the YSS group. Glutathione S-transferase (GST) further enhances the antioxidant capacity by catalyzing the binding of glutathione to electrophilic substrates [28], which was upregulated 1.18-fold in YSS. Therefore, sesamol treatment improved the total antioxidant capacity of *Aurantiochytrium* sp. DECR-KO, which was beneficial for accumulating more PUFAs.

### 2.6. Detection of the Gene Expression through qRT-PCR

**Figure 5:**
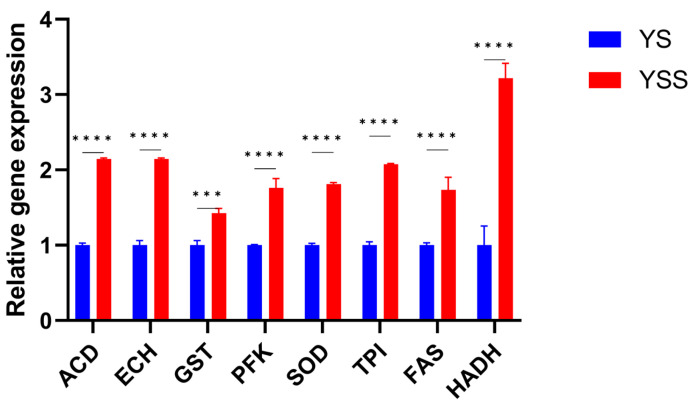
The relative expression levels of related enzyme genes by qRT-PCR. *** *p* < 0.001, and **** *p* < 0.0001.

The expression profiles of eight genes related to fatty acid synthesis were analyzed to validate the transcriptome analysis data (Figure 5). The results of reverse transcriptase quantitative PCR (RT-qPCR) were consistent with the transcriptome sequencing (RNA-Seq) results.

## 3. Discussion

### 3.1. Effect of Fermentation Optimization for Growth

The results of elemental N content showed a different trend from that of biomass concentration obtained from culture, which may be related to the solubility rate of each organic nitrogen source, and the efficiency of its availability to *Aurantiochytrium* sp. DECR-KO. The phenomenon that peptone had the highest N content but the least biomass concentration obtained from culture may be due to the fact that peptone was least consistent with the amino acid composition of *Aurantiochytrium* sp. DECR-KO, and it is difficult to be utilized by *Aurantiochytrium* sp. DECR-KO in the pre- and mid-fermentation stages, which led to its accumulation of less biomass concentration [29].

Medium composition and fermentation conditions significantly affect fatty acid accumulation in *Aurantiochytrium*. Influential factors include the selection of carbon and nitrogen sources, culture strategy, dissolved oxygen concentration, salinity, pH and temperature [30]. To improve the lipid production capacity of *Aurantiochytrium*, a thorough evaluation of the effects of different fermentation conditions on it is required. The *Aurantiochytrium*’s development and metabolism are significantly impacted by temperature. The temperature between 20 and 30 °C seems to be the optimum incubation temperature [31]. Low temperature had been shown to stimulate DHA production to maintain membrane fluidity and permeability, but at the expense of biomass, resulting in lower overall DHA production [32]. *Aurantiochytrium* is found from mangroves and other sea areas, while the average salinity of natural seawater is 35‰, and the optimal salinity for growth and tolerance level varies according to the strains [33]. High salinity stress can stimulate lipid accumulation in thraustochytrids [32], while high salinity can corrode equipment and increase costs. In *Schizochytrium limacinum* OUC88, the lipid content and biomass was significantly reduced when the salinity was less than 18 g/L (51% of seawater). Both inorganic (such as nitrate and ammonium) and organic nitrogen (such as yeast extract, peptone and corn steep liquor) can be utilized by *Aurantiochytrium* [34,35,36]. The combination of organic nitrogen proved to be more supportive of production because of the non-specific growth factors (vitamins, trace elements) it provided [15]. For thraustochytrids, balancing the relationship between biomass concentration and lipid content per cell is important to increase the total lipid yield. A single increase in lipid content may lead to a reduction in biomass concentration [17]. Therefore, a staged culture strategy was used to separate the biomass increase stage from the lipid accumulation stage, increasing the final lipid yield. Response surface methodology allows for the identification of optimal culture conditions that will improve the growth of *Aurantiochytrium* [37]. In this study, the optimal conditions for maximum growth under the first phase were determined by response surface methodology optimization.

### 3.2. The Effect of Sesamol Additionon Lipid Accumulation

Sesamol is a naturally occurring phenolic molecule that is added to foods and medicines as a cheap and safe antioxidant [38]. Despite the antioxidant activity of sesamol, the presence of 0.5 mM sesamol decreased the fatty acids content of *Crypthecodinium cohnii* by 25.24% [39]. In *Schizochytrium* sp., the addition of 1 mM sesamol caused a 59.06% increase in lipid yield [22]. The fatty acid content of *Aurantiochytrium* was also significantly reduced in this study by 0.5 mM sesamol. When sesamol was added at a concentration greater than 1 mM, the yield of total fatty acids increased with increasing concentration. Compared to other oil-producing microorganisms, sesamol induces lipid synthesis in *Aurantiochytrium* sp. possibly due to the presence of a specific fatty acid synthesis system in *Aurantiochytrium* sp.

**Figure 6:**
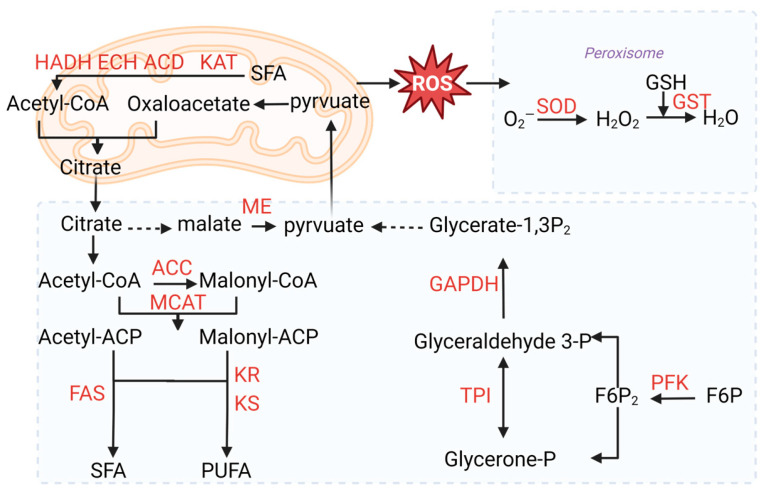
Schematic map of transcriptional analysis of the pathways associated with lipid and carbon metabolism in *Aurantiochytrium* sp. DECR-KO. HADH: 3-hydroxyacyl-CoA dehydrogenase; ECH: enoyl-CoA hydratase; ACD: acyl-CoA dehydrogenase; KAT: 3-ketoacyl-CoA thiolase; SOD: superoxide dismutase; GST: glutathione S-transferase; ACC: acetyl-CoA carboxylase; MCAT: malonyl-CoA:ACP transacylase; ME: malic enzyme; FAS: fatty acid synthase; KS: 3-ketoacyl-synthase; KR: ketoreductase; PFK: 6-phosphofructokinase; TPI: triosephosphate isomerase; GAPDH: glyceraldehyde 3-phosphate dehydrogenase.

Two independent pathways for polyunsaturated fatty acid (PUFA) synthesis pathways were reported in *Aurantiochytrium*. In the fatty acid synthase (FAS) pathway, firstly in the action of fatty acid synthase, acetyl-CoA and malonyl-CoA are used to produce palmitic acid (C16:0), and then PUFAs are produced from C16:0 through a sequence of desaturases and elongases [40]. In the polyketide synthase (PKS) pathway, PUFAs can be generated more efficiently starting from acetyl-ACP without oxygen dependence [41]. In fatty acid production, NADPH is the crucial precursor in the FAS and PKS pathway [12]. ME (malic enzyme) and G6PD(glucose-6-phosphate 1-dehydrogenase) are crucial enzymes in the production of NADPH. The overexpression of G6PD and ME increased NADPH supply, resulting in a 10.6% and >105% increase in PUFA and SFA, respectively [42,43]. Sesamol can reduce NADPH supply by inhibiting ME, leading to a reduction in lipid accumulation in oleaginous microorganisms [44]. Previous studies have shown that the addition of sesamol leads to a decrease in ME activity and an increase in G6PD activity in *Schizochytrium* sp.H016 [22]. However, there was no significant difference in the expression of G6PD by the addition of sesamol in the present study, while ME was upregulated 1.00-fold in the YSS group. The increase in ME expression may have compensated for the decrease in its activity to ensure the supply of NADPH. For the production of another precursor, acetyl-CoA, there was no ATP citrate lyase (ACL) identified in this study. In the FAS pathway, fatty acid synthase was upregulated, leading to the accumulation of SFA (Figure 6). In the PKS pathway, the synergistic activity of β-ketoacyl synthase (KS), β-ketoreductase (KR), dehydration, and enoyl-reductase (ER) lead to the synthesis of PUFA [45]. The overexpression of PKS pathway genes increased the accumulation of DHA in the YSS group. Interestingly, genes associated with fatty acid degradation were significantly upregulated. The β-oxidation of fatty acids is generally considered to be detrimental to fatty acid accumulation. However, β-oxidation provides acetyl coenzyme A and ATP, which are also necessary for the synthesis of fatty acid. The upregulation of the fatty acid degradation pathway may result from the consumption of large amounts of short-chain fatty acids for cell division and other life activities [46]. Acetyl coenzyme A generated from the breakdown of short-chain fatty acids can enter the TCA cycle or serve as a precursor substance for unsaturated fatty acids. PUFAs have a high degree of unsaturation, which makes them easily oxidized. Due to the increased accumulation of polyunsaturated fatty acids, there is an increased risk of lipid peroxidation, which is accompanied by increased levels of ROS [47]. In this study, the antioxidant system mitigates oxidative stress damage through enzymatic (superoxide dismutase) and non-enzymatic mechanisms (glutathione S-transferase) [48]. Superoxide dismutase (SOD) can catalyze superoxide anions, the precursors of most ROS, to oxygen and hydrogen peroxide. Glutathione S-transferase (GST) quenches reactive molecules by adding glutathione, assisting in the elimination of hydrogen peroxide and other oxidative stress metabolites [49].

## 4. Materials and Methods

### 4.1. Nitrogen Content Determination

The nitrogen elemental composition of six nitrogen sources (yeast extract, bacteriological peptone, cottonseed powder, peanut powder, corn powder, and soybean powder) was determined using an elemental analyzer and sulfanilamide as a standard.

### 4.2. Strain and Cultural Methods

The *Aurantiochytrium* sp. DECR knockout engineered strain (*Aurantiochytrium* sp. DECR-KO) is stored in the China Center for Type Culture Collection (CCTCC M 2022545) and obtained from prior study [23,50]. Routine culture conditions are as follows: M4 culture medium (1 g/L yeast extract, 20 g/L glucose, 0.025 g/L potassium dihydrogen phosphate and 1.5 g/L peptone dissolved in artificial seawater with a salinity of 30‰), 23 °C and 200 rpm. The strain was cultured in a 250 mL shake flask with 100 mL M4 culture medium.

### 4.3. Determination of Cell Dry Weight and Neutral Lipids

Cultured cells were collected by centrifugation at 8000 rpm for 10 min and then freeze-drying using a freeze-dryer for 48 h. The cell dry weight (DCW) was used as the biomass.

Neutral lipids were stained with Nile red fluorescent dye (Rhawn, Shanghai, China). First, 0.1 mg/mL Nile red (in acetone) is used for staining 200 mL of cells. After 20 min of a dark incubation at 200 rpm, Nile red fluorescence was measured using a fluorescence microplate reader (Synergy H1, Bio-Tek, Winooski, VT, USA) with an excitation wavelength of 488 nm and emission wavelength of 592 nm [51].

### 4.4. Experimental Design

To maximize the biomass production of *Aurantiochytrium* sp. DECR-KO by optimizing culture conditions, this study selected four factors (temperature, salinity, nitrogen concentration and nitrogen ratio) to carry out a one-way experimental design to observe their effects on the biomass production. *Aurantiochytrium* sp. DECR-KO was inoculated into 250 mL shake flasks with 100 mL of M4 medium and incubated at 23 °C and 200 rpm for 42 h as a seed culture. For temperature, the seed culture was inoculated into M4 medium at six temperatures: 17 °C, 20 °C, 23 °C, 26 °C, 29 °C, and 32 °C. For salinity, seed culture was inoculated into M4 medium with different salinities (0‰, 10‰, 20‰, 30‰, 40‰, 50‰) at 23 °C. The nitrogen source concentration of the M4 medium was calculated to be about 0.31 g/L by the percentage of N content. For nitrogen concentration, the medium with different nitrogen source concentrations (0.1, 0.3, 0.5, 0.7, 0.9, and 1.1 g/L) was prepared at a ratio of 1:1.5 between yeast extract and soya bean powder, respectively. The seed culture was inoculated into M4 medium with different concentrations of nitrogen sources at 23 °C. The medium with different ratios of yeast extract and soybean flour (3:1, 2:1, 1:1, 1:1.5, 1:2, 1:3) was prepared separately. Seed culture was inoculated into M4 medium with different nitrogen source ratios at 23 °C. All experiments were inoculated into 250 mL culture flasks containing 100 mL of medium and incubated at 200 rpm with shaking for 42 h. Cells were collected by centrifugation and weighed after freeze-drying. Then, we carried out a Box–Behnken design (BBD) of experiments based on the results and finally obtained the optimized culture conditions with the highest biomass production.

A Box–Behnken experimental design with four factors, namely, temperature (A), salinity (B), nitrogen concentration (C) and nitrogen ratio (D), as independent variables, with −1, 0, and +1 levels for each factor, and cell dry weight (DCW) as the response value was carried out as shown in Table 4. The experimental design was assisted by Design-Expert 12 software. The design of the central experimental site was 5, which required a total of 29 experiments, and each experiment was incubated for 42 h. The experimental results were entered into Design-Expert software for response surface analysis.

DMSO and ethanol were used as solvents, and 1‰ and 0.5‰ (*v*/*v*) of different solvents were added to the strain culture medium that had been cultured for 42 h. The dry cell weight and Nile red relative fluorescence intensity were determined after 24 h of incubation. A gradient concentration of 0, 0.5, 1, 1.5, 2, and 2.5 mM sesamol was supplemented into the *Aurantiochytrium* sp. DECR-KO that had been cultured for 42 h in optimized culture conditions (temperature 28.7 °C, 11.62 g/L artificial sea salt (salinity of 10.7‰), 3.16 g/L yeast extract, 1.58 g/L water-soluble soybean powder, 20 g/L glucose, 0.025 g/L potassium dihydrogen phosphate). The culture was continued under optimized culture conditions for 21 h to stationary phase (63 h) [23].

### 4.5. Lipid Extraction and Fatty Acid Analysis

Lipids were extracted by a chloroform–methanol (2:1, *v*/*v*) method as previously described [52,53]. First, 500 mg of the freeze-dried cells was mixed with chloroform–methanol and extracted for 72 h at 65 °C in a Soxhlet extractor (AG-SXT-06, OUGE, Shanghai, China). The crude total lipids were obtained by evaporating the solvent at 65 °C. To the crude total lipids, 4 mL of 4% sulfuric acid in methanol was added to obtain fatty acid methyl esters (FAMEs) at 65 °C for 1 h. The FAMEs were treated with hexane and deionized water, which was followed by volatilization of the hexane off in a stream of nitrogen to gain the methyl esterified fatty acids (MEFs). Then, 1 mL of dichloromethane was used for the dissolution of the MEFs. Compositional and content analyses of MEFs were performed by gas chromatography–mass spectrometry (GC-MS, 7890-5975 Agilent, Santa Clara, CA, USA). Chromatographic conditions were set as claimed in previous studies [53]. The mass spectrometry library of the National Institute of Standards and Technology (NIST) was used to identify the fatty acids. Methyl nonadecylate (Solarbio, Beijing, China) was used as an internal standard, and the content was determined by comparing the internal standard peak areas.

### 4.6. RNA Extraction, Transcriptomic Analysis, and Real-Time Quantitative PCR (RT-qPCR) Analysis

For transcriptome analysis, samples were collected at 63 h for RNA extraction. Then, samples were sent to the BioTechnology Genomics Institute, Shenzhen, China for transcriptome sequencing. Under the condition of fold change ≥ 2 and adjusted *p*-value ≤ 0.001, DEseq2 was used to conduct differential gene analysis between groups [54]. According to the gene ontology (GO) and Kyoto Encyclopedia of Genes and Genomes (KEGG) annotation results and official classification, the differentially expressed genes were functionally classified, and the phyper in R software was used for KEGG enrichment analysis. Genes satisfying Q-value ≤ 0.05 were defined as significantly enriched.

The total RNA was extracted and collected from both the experimental and control sample using a Trizol reagent. RNA concentration and purity measurements were performed by NanoDrop2000 (Thermo Scientific, Waltham, MA, USA). The PrimeScript™ RT reagent Kit was used for cDNA synthesis. According to the manufacturer’s protocol, TB Green^®^ Premix Ex Taq™ II and ABI QuantStudio 6 Flex (Applied Biosystems, Foster, CA, USA) were used for RT-qPCR. Primer sequences used for RT-qPCR (Table S2) were designed by Primer Premier 5.0. The relative gene expression was calculated as 2^−ᐃᐃct^ using 18S rDNA as the internal standard [26].

### 4.7. Statistical Analysis

All the experimental data were expressed as the mean ± standard deviation (S.D.) of at least three independent experiments. Design Expert 12 software was used to perform response surface experiments. GraphPad Prism (version 8.0.2) was used to analyze data. Two-way ANOVA and *t*-tests were used to determine differences between groups at a confidence level of *p* < 0.05. A different number of asterisks (*) on each column indicates the significance of the difference, * *p* < 0.05, ** *p* < 0.01, *** *p* < 0.001, and **** *p* < 0.0001.

## 5. Conclusions

In the present study, the lipid production of *Aurantiochytrium* sp. DECR-KO was enhanced by a two-phase strategy. In the first stage, the biomass concentration of *Aurantiochytrium* sp. DECR-KO was significantly increased by response surface methodology optimization. In the second stage, the fatty acid yield was increased by adding the antioxidant sesamol. Compared to the M4 culture condition, the biomass concentration, total fatty acid yield and DHA yield of 2.5 mM sesamol treatment were increased by 49.85%, 93.49% and 98.22%, respectively. The treatment of sesamol induced the gene expression related to fatty acid synthesis (FAS, KS, KR) and the antioxidant system (SOD, GST). This research provides a methodological basis for the use of *Aurantiochytrium* sp. DECR-KO as a feedstock for the industrial production of DHA.

## Figures and Tables

**Table 1:** The Box–Behnken design matrix for real values and coded values (in parentheses).

Run	A: Temperature (°C)	B: Salinity (%)	C: N Ratio	D: N Concentration (g/L)	DCW (g/L)
1	26 (−1)	10 (0)	3 (+1)	0.9 (0)	5.12
2	29 (0)	20 (+1)	1 (−1)	0.9 (0)	5.20
3	32 (+1)	10 (0)	1 (−1)	0.9 (0)	4.92
4	29 (0)	0 (−1)	2 (0)	0.7 (−1)	4.73
5	29 (0)	10 (0)	2 (0)	0.9 (0)	5.75
6	29 (0)	20 (+1)	3 (+1)	0.9 (0)	4.79
7	29 (0)	10 (0)	2 (0)	0.9 (0)	5.49
8	29 (0)	0 (−1)	2 (0)	1.1 (+1)	5.17
9	29 (0)	0 (−1)	1 (−1)	0.9 (0)	4.58
10	29 (0)	20 (+1)	2 (0)	0.7 (−1)	4.90
11	26 (−1)	10 (0)	2 (0)	1.1 (+1)	5.33
12	29 (0)	10 (0)	2 (0)	0.9 (0)	5.69
13	32 (+1)	0 (−1)	2 (0)	0.9 (0)	4.22
14	29 (0)	10 (0)	2 (0)	0.9 (0)	5.49
15	29 (0)	10 (0)	3 (+1)	1.1 (+1)	5.79
16	32 (+1)	10 (0)	2 (0)	1.1 (+1)	4.92
17	26 (−1)	0 (−1)	2 (0)	0.9 (0)	4.78
18	29 (0)	0 (−1)	3 (+1)	0.9 (0)	4.59
19	29 (0)	10 (0)	1 (−1)	0.7 (−1)	5.03
20	29 (0)	20 (+1)	2 (0)	1.1 (+1)	5.01
21	32 (+1)	10 (0)	3 (+1)	0.9 (0)	5.44
22	29 (0)	10 (0)	2 (0)	0.9 (0)	5.65
23	26 (−1)	20 (+1)	2 (0)	0.9 (0)	4.94
24	29 (0)	10 (0)	3 (+1)	0.7 (−1)	5.17
25	32 (+1)	20 (+1)	2 (0)	0.9 (0)	4.86
26	26 (−1)	10 (0)	1 (−1)	0.9 (0)	5.47
27	26 (−1)	10 (0)	2 (0)	0.7 (−1)	5.17
28	32 (+1)	10 (0)	2 (0)	0.7 (−1)	4.99
29	29 (0)	10 (0)	1 (−1)	1.1 (+1)	5.38

**Table 2:** ANOVA analysis of response surface experiment.

Source	Sum of Squares	df	Mean Square	*F*-Value	*p*-Value	
Model	3.5100	11	0.3191	9.45	<0.0001	significant
A—temperature	0.1757	1	0.1757	5.20	0.0357	
B—salinity	0.2172	1	0.2172	6.43	0.0213	
C—the ratio of two nitrogen sources	0.0085	1	0.0085	0.2508	0.6229	
D—nitrogen source concentration	0.2184	1	0.2184	6.47	0.0210	
AB	0.0589	1	0.0589	1.74	0.2042	
AC	0.1888	1	0.1888	5.59	0.0302	
AD	0.0123	1	0.0123	0.3646	0.5539	
A^2^	0.6196	1	0.6196	18.34	0.0005	
B^2^	2.3700	1	2.37	70.27	<0.0001	
C^2^	0.1335	1	0.1335	3.95	0.0631	
D^2^	0.1073	1	0.1073	3.18	0.0926	
Residual	0.5742	17	0.0338			
Lack of Fit	0.5176	13	0.0398	2.82	0.1640	not significant
Pure Error	0.0566	4	0.0141			
Cor Total	4.0800	28				

**Table 3:** Transcriptomics of the expression of genes under sesamol treatment.

Gene ID	Name	Description	YSS vs. YS (log_2_ Fold Change)
Fatty Acid Synthesis			
TRINITY_DN338_c0_g1_i2-SM4	ACC	acetyl-CoA carboxylase	0.81
TRINITY_DN7076_c0_g1_i1-SM4	MCAT	malonyl-CoA:ACP transacylase	1.07
TRINITY_DN14219_c0_g1_i1-SM4	ME	malate dehydrogenase (oxaloacetate-decarboxylating)	1.00
TRINITY_DN11008_c0_g1_i1-YS	FAS	fatty acid synthase	0.92
TRINITY_DN15725_c0_g1_i1-YSS	KS	3-ketoacyl-synthase	0.68
TRINITY_DN4969_c0_g2_i1-YS	KR	ketoreductase	1.33
TRINITY_DN2556_c0_g1_i1-YSS	PFK	6-phosphofructokinase	1.07
TRINITY_DN897_c2_g1_i1-YSS	TPI	triosephosphate isomerase	1.00
TRINITY_DN10506_c0_g1_i1-YSS	GAPDH	glyceraldehyde 3-phosphate dehydrogenase	1.21
Fatty Acid Degradation			
TRINITY_DN2005_c6_g1_i1-YSS	HADH	3-hydroxyacyl-CoA dehydrogenase	1.45
TRINITY_DN13554_c0_g1_i1-AM4	ECH	enoyl-CoA hydratase	1.38
TRINITY_DN11028_c0_g1_i1-YS	ACD	acyl-CoA dehydrogenase	1.17
TRINITY_DN2439_c0_g1_i1-YSS	KAT	3-ketoacyl-CoA thiolase	0.90
antioxidant system			
TRINITY_DN12527_c0_g1_i1-YSS	GST	glutathione S-transferase	1.18
TRINITY_DN11074_c0_g1_i1-AM4	SOD	superoxide dismutase	1.27

**Table 4:** Factors and levels of Box–Behnken for the optimization of the culture conditions of the *Aurantiochytrium* sp. DECR-KO.

	A	B	C	D
Level	Temperature (°C)	Salinity (‰)	nitrogen concentration (g/L)	nitrogen ratio *
−1	26	0	0.7	1:1
0	29	10	0.9	2:1
1	32	20	1.1	3:1

* The nitrogen ratio is the weight ratio of yeast extract to water-soluble soybean powder.

## Data Availability

The data presented in this study are available on request from the corresponding author.

## Supplementary Materials

The following supporting information can be downloaded at https://www.mdpi.com/article/10.3390/md22080371/s1, Figure S1: Differential gene KEGG pathway enrichment bubble map; Table S1: Nitrogen content of different nitrogen sources; Table S2: The primer pairs used in the cloning experiment.
