# Supplementary material for: Comparative Transcriptomic Analysis on the Effect of Sesamol on the Two-Stages Fermentation of Aurantiochytrium sp. for Enhancing DHA Accumulation
Source: Mar Drugs. 2024 Aug 16;22(8):371. doi: 10.3390/md22080371 (PMC11355499; doi:10.3390/md22080371)
Supplement: Supplementary file 1 [file marinedrugs-22-00371-s001.zip › Table S1.docx]

Table S1 Nitrogen content of different nitrogen sources

| nitrogen sources | Elemental N determination（%） |
| --- | --- |
| peptone | 13.35±0.08 |
| Yeast extract | 10.80±0.08 |
| Water-soluble peanut powder | 11.43±0.48 |
| Water-soluble cottonseed powder | 10.93±0.37 |
| Water-soluble soybean powder | 10.23±0.30 |
| Water-soluble corn protein powder | 11.36±0.33 |
