# Supplementary material for: Comparative Transcriptomic Analysis on the Effect of Sesamol on the Two-Stages Fermentation of Aurantiochytrium sp. for Enhancing DHA Accumulation
Source: Mar Drugs. 2024 Aug 16;22(8):371. doi: 10.3390/md22080371 (PMC11355499; doi:10.3390/md22080371)
Supplement: Supplementary file 1 [file marinedrugs-22-00371-s001.zip › Table S2.docx]

Table S2 The gene specific primers of RT-qPCR

| Gene name | Sequences(5’-3’) | ID | Product Length (bp) |
| --- | --- | --- | --- |
| KR | F:GCCACCATCTGCTGCGAGTAAT  R:ATGACCAAGGTTTCCGCCGATG | TRINITY_DN4969_c0_g2_i1-YS | 180 |
| PFK | F:ATGGCACGCTCACAATCGGTAC  R:TGGCTCCTTCGGCAACAAGAAC | TRINITY_DN2556_c0_g1_i1-YSS | 291 |
| TPI | F:AGCACCGACCATGATCTCAGAG  R:TCGCCAGGACATCCAAGAACTT | TRINITY_DN897_c2_g1_i1-YSS | 279 |
| GST | F:ATGGTGCCTCCCTGAGAGTTGA  R:GCGTGCGTCTGAATAGTTGCTT | TRINITY_DN12527_c0_g1_i1-YSS | 208 |
| SOD | F:GCCGTGCTCATCATCAGAGACA  R:GCATCACTGAAGAGGCGAACTG | TRINITY_DN11074_c0_g1_i1-AM4 | 191 |
| HADH | F:CGAGGCAGCAACCAAGACTTCA  R:GCACCACGCTCCGAGAAGAATA | TRINITY_DN2005_c6_g1_i1-YSS | 123 |
| ACD | F:TGAGGCTGATGGCGATGACTAC  R:ATGGACTGCTGCTGGTGAAGAG | TRINITY_DN11028_c0_g1_i1-YS | 135 |
| ECH | F:TGGTCTACGCTGATCCGCTTTC  R:TTGAACGCCGCATCTTCCACAG | TRINITY_DN13554_c0_g1_i1-AM4 | 212 |
| FAS | F:ATCCGCCAGCACGACAACAT  R:TGACAACACCACCAGCAACCTC | TRINITY_DN11008_c0_g1_i1-YS | 277 |
