# Supplementary figures and images for: Comparative Transcriptomic Analysis on the Effect of Sesamol on the Two-Stages Fermentation of Aurantiochytrium sp. for Enhancing DHA Accumulation
Source: Mar Drugs. 2024 Aug 16;22(8):371. doi: 10.3390/md22080371 (PMC11355499; doi:10.3390/md22080371)

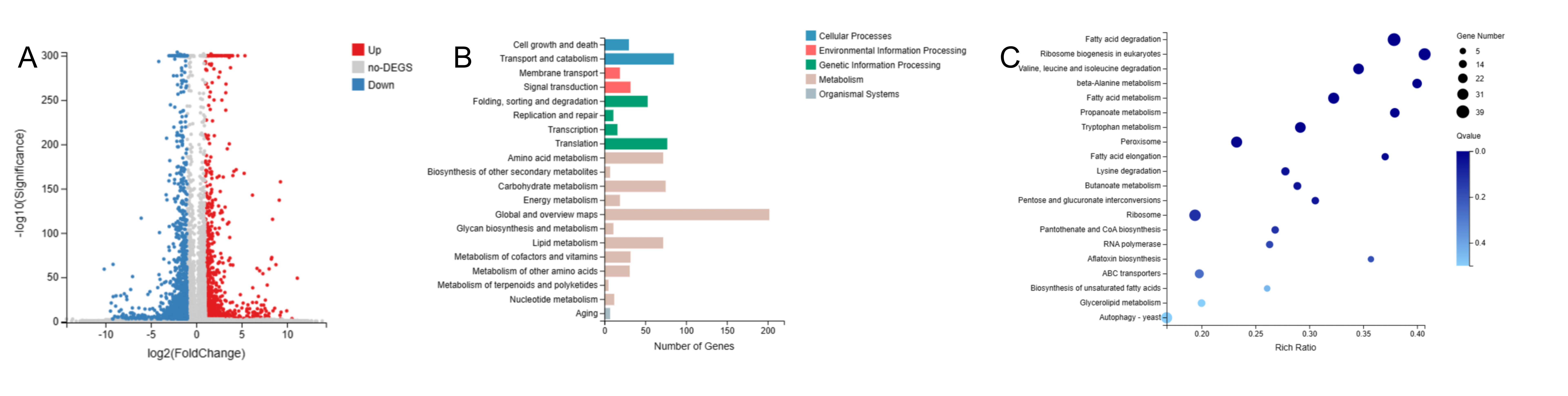

Supplement: Supplementary file 1 [file marinedrugs-22-00371-s001.zip › figure S1.png]
